# Practical Medicine

**Published:** 1876-02

**Authors:** 


### III. Practical Medicine.

1. *Strangulation of a Loop of the Ileum by a Band of the Mesentery.* Judson Bradley, M.D. (*Detroit Review*, January, 1876.)

A girl six years old, having previously enjoyed good health, was attacked suddenly after dinner, with pain in the abdomen. With all the symptoms of intestinal obstruction the child lingered ten days and died of inanition.

*Post-mortem*, there was found no intussusception, but a knot of ileum "bound by a band of what, on closer inspection, proved to be a portion of the mesentery, holding in a peculiar manner about two feet of ileum so closely that nothing could pass." The mesenteric glands were enlarged, but there was no other pathological condition. The knot was about one foot from the cœcum, "and close to the spinal column on the right side." The author is at loss to account for the condition discovered.

[Is Dr. B. sure the child had not months before had a localized inflammation in the abdomen?—Ed.]

2. *Local Treatment of Chronic Dysentery.* T. Gaillard Thomas, M.D. (*N. Y. Med. Jour.*, January, 1876.)

A case is here reported of chronic dysentery of five years' standing, following an acute attack, which had resisted about all the known or frequently used forms of treatment, administered by all physicians. The patient was a woman, who stated that during the five years of its existence acute attacks had been frequently engrafted on the chronic malady, apparently excited by indiscretions in diet or unusual fatigue. The smallest number of dejections daily, during the whole period was 8, but it was not unusual for 27 or more to occur. At the time the local treatment was begun she was in a "desperate condition" of debility. Dr. T. placed the patient in the left lateral position and under an anæsthetic, and, with the aid of a long duck-bill speculum and a retractor, explored the rectum to the sigmoid flexure. Washing off the mucous membrane it "was seen swollen, œdematous, hanging in hæmorrhoidal masses, and studded with deep ulcers with grayish bottoms. It was of a deep red, almost violet hue."

At a subsequent examination of the same character, from a swab of wet cotton on a whalebone rod, all the ulcers from the sigmoid flexure to the anus were lightly touched with commercial nitric acid. The cauterization was carefully graduated, so that the possibility of any sloughing was avoided.

Only a little pain was experienced on recovering from the anæsthetic, and the patient declared afterward that this gave her the first real respite she had had for five years. Two other similar applications were made at the interval of a week each, and the patient was practically cured. She soon returned to her home and declared herself well. Rest and the milk diet were insisted on during the treatment.

Dr. T. thinks, with care to avoid such cauterization as to produce sloughing, there is no danger of rectal stricture from the use of nitric acid. This treatment originated with Dr. R. B. Maury, of Memphis, Tenn., who had treated twelve cases, eleven of which recovered. The caustic he used, however, was nitrate of silver, in strength from the solid stick to that of a solution of a drachm to the ounce of water. He thinks the caustic is beneficial not only in stimulating the healing of the ulcers but in blunting the sensibility of the irritable rectum, thus restoring quiet to the whole intestinal tract.

3. *The Pathology of Follicular Disease of the Throat and Air Passages.* BEVERLY ROBINSON, M.D. (*Am. Jour. Med. Sciences*, January, 1876.)

This disease—the clergyman's sorethroat—is here held to be a follicular inflammation, and is due to a diathesis or constitutional tendency. The cases are characterized locally by "granulations" on the pharynx, velum, pillars of the fauces and tonsils. These are small glistening bodies, often not larger than a pin's head, and resembling herpetic vesicles covered by mucus. Sometimes there are unpleasant sensations in swallowing and more or less hoarseness and aphonia; sometimes neither of these. No constitutional character, other than that mentioned, specially predisposes to the disorder, nor does the work of public speaking necessarily do this.

Dr. R. believes that at the beginning of an attack there is an infiltration and thickening of the membrane, and that later there is an atrophic degeneration of the tissues—the mucous membrane, the submucous tissue and the muscular fibres underlying the membrane—by which there is an increase in the antero-posterior diameter of the pharynx. When the glands of the pharynx become ulcerated, as they frequently do, he believes it due to a want of nerve force from an interstitial *neuritis* of the nerves supplying the part. This neuritis explains also the symptoms of pain and disagreeable sensations in the throat, as well as the aphonia without laryngeal lesion, so frequently attendant on this disorder.

Local treatment alone is not competent to cure this affection—general treatment, as well, is always required.

4. *Post-nasal Catarrh.* BEVERLY JOHNSON, M.D. (Read before N. Y. Co. Med. Society, Sept. 27, 1875.)

Dr. Johnson thinks the disorder under consideration is little understood especially as to its pathology. Post-nasal catarrh is not ordinary rhinitis; it is not acute or chronic coryza, however the latter may predispose to it, but it is chronic *follicular* disease of the nasal and naso-pharyngeal cavities. He believes it to be merely a local determination of a diathetic state, and that it differs nowise in nature from chronic follicular disease of the throat and remaining portions of the air passages.

Follicular disease of the naso-pharyngeal space has two constant symptoms: 1st, a sensation of fullness and *stuffiness* in the region affected; and 2nd, the falling down from above the palate and posterior nares of particles of mucus, varying in physical characters. No other symptoms are pathognomonic. Sometimes this mucus may be seen trickling down the posterior wall of the pharynx. By the rhinoscopic mirror at times it may be seen hanging like stalactites from the roof of the pharynx. In advanced cases masses of this mucus become inspissated, thick and hard, and irritating the part produce ulcers and fetor. Ulcers are found on the septum, roof of the posterior nares and vault of the pharynx, and occasionally on the turbinated bones and walls of the nasal fossæ.

Systemic treatment is of first importance—iron, quinine, cod-liver oil, arsenic and strychnia for tonics. Such medicaments as, given internally, exert a beneficial effect on the glands and mucous lining of the throat and nose, are next of value.

Of medicines which act in the last mentioned way—when patients are free from any other diathesis, as herpetic, gouty, syphilitic, scrofulous and tubercular—he classes as most valuable, sulphur, cubebs and ammoniacum. The first he administers in the water of the white sulphur springs of Sharon, a tumblerfull three times a day. Of cubebs he gives fifteen grains of the powder, in a mixture, every two or three hours, for a number of days; and, after a few days of rest, resuming it again. Ammoniacum he gives in doses of j—iij grs. Local treatment is not so efficacious as usually supposed. The douche has been greatly abused, by being used with too cold solutions and too vigorously; great damage has been done to the organs of smell and hearing.

Solutions applied to the diseased part are usually too weak—yet there is great danger of making them too strong. The preferable local medication is by spray and powders. The spray should be used simply warm. The best formula is this: R. Acidi Carbol. liq. m xi; Boracis, Sodii bicarb. *aa* dr. ij; Glycerini, dr. vij; Aquam ad. oz. viij. Mix. Used morning and evening for five minutes by means of a hard rubber atomizer. Of powders to be thrown up behind the palate by a powder blower, he gives preference to one made of equal parts of powdered iodoform and camphor and two to four times the amount of powdered acacia—to be used one or more times daily.

5. *Pulmonary Collapse.* H. A. DUBOIS, M.D. (*Trans. Med. Soc. California*, 1874–5.)

The writer of this paper believes that in many cases of so-called lobular pneumonia in children, there is nothing but collapse of portions of the lung superadded to a bronchitis, either mild or severe. When in the midst of a bronchitis there occurs a sudden and permanent increase in the frequency of respiration without an apparent exaggeration of the inflammation, he believes the condition is quite sure to be one of collapse. Auscultation and percussion are of very small diagnostic value.

An illustrative case is recorded. A child of five years old had some obscure ailment that was guessed to be malarial; soon the breathing became rapid and symptoms of bronchitis slowly supervened. Shortly the respirations became 50—60 per minute, afterward they reached 80, 100, and finally 130, while the "pulse was never over 120—130 and frequently much lower, and a temperature never higher than F. 102° and often as low as F. 97.5°." For five months this rapid respiration had continued—long after all evidence of inflammation had disappeared.

All expectorants and depressing measures did injury; only food and tonics and stimulation were of any value. Dr. Dubois believes in all such cases the last mentioned treatment should be resorted to.

6. *Encephaloid Disease of the Lung.* G. G. TYRRELL, M.D. (*Trans. Med. Soc. Cal.*, 1874-5.)

A case is here reported of this disorder in a girl of fourteen, who had been previously healthy, developed insidiously and slowly at first, being such as might attend incipient phthisis It was only seven weeks before the fatal termination of the case that the child was taken out of school because it was supposed she was studying too hard. A month later, when professional aid was called, the girl was able to walk about, had no subjective symptoms but shortness of breath and slight weakness. On examination, the left chest was found almost perfectly solid. The heart was crowded to the right side.

The growth developed rapidly, a tumor the size of a large cocoanut appearing under the arm, and death occurring by asphyxia in three weeks. Exploration of this tumor a few days before death, with the expectation of finding it an accumulation of fluid in the pleura, discovered it to be an encephaloid mass involving the lung.

